# The Role of a Conserved Arg-Asp Pair in the Structure and Function of Tetanus Neurotoxin

**DOI:** 10.3390/toxins17060273

**Published:** 2025-05-30

**Authors:** Elizabeth A. Wilson, Ashtyn N. Bevans, Michael R. Baldwin

**Affiliations:** Department of Molecular Microbiology and Immunology, School of Medicine, University of Missouri, Columbia, MO 65212, USA; wilsoneli@missouri.edu (E.A.W.); anb8mf@health.missouri.edu (A.N.B.)

**Keywords:** tetanus toxin, translocation, clostridial neurotoxins, pore formation, pH trigger

## Abstract

Tetanus, a severe and life-threatening illness caused by *Clostridium tetani*, produces symptoms such as muscle spasms, muscle stiffness and seizures caused by the production of tetanus neurotoxin (TeNT). TeNT causes spastic paralysis through the inhibition of neurotransmission in spinal inhibitory interneurons. This is achieved, in part, through pH-triggered membrane insertion of the translocation (HCT) domain, which delivers the catalytic light-chain (LC) domain to the cytosol. While the function of HCT is well defined, the mechanism by which it accomplishes this task is largely unknown. Based on the crystal structure of tetanus neurotoxin, we identified potential polar interactions between arginine 711, tryptophan 715 and aspartate 821 that appear to be evolutionarily conserved across the clostridial neurotoxin family. We show that the disruption of the Asp-Arg pair in a beltless HCT variant (bHCT) results in changes in thermal stability without significant alterations to the overall secondary structure. ANS (1-anilino-8-napthalene sulfonate) binding studies, in conjunction with liposome permeabilization assays, demonstrate that mutations at R711 or D821 trigger interactions with the membrane at higher pH values compared to wildtype bHCT. Interestingly, we show that the introduction of the D821N mutation into LH_N_T (LC-HCT only), but not the holotoxin, resulted in the increased cleavage of VAMP 2 in cortical neurons relative to the wildtype protein. This suggests that, as observed for botulinum toxin A, the receptor-binding domain is not necessary for LC translocation but rather helps determine the pH threshold of membrane insertion. The mutation of W715 did not result in detectable changes in the activity of either bHCT or the holotoxin, suggesting that it plays only a minor role in stabilizing the structure of the toxin. We conclude that the protonation of D821 at low pH disrupts interactions with R711 and W715, helping to drive the conformational refolding of HCT needed for membrane insertion and the subsequent translocation of the LC.

## 1. Introduction

**Figure 1:**
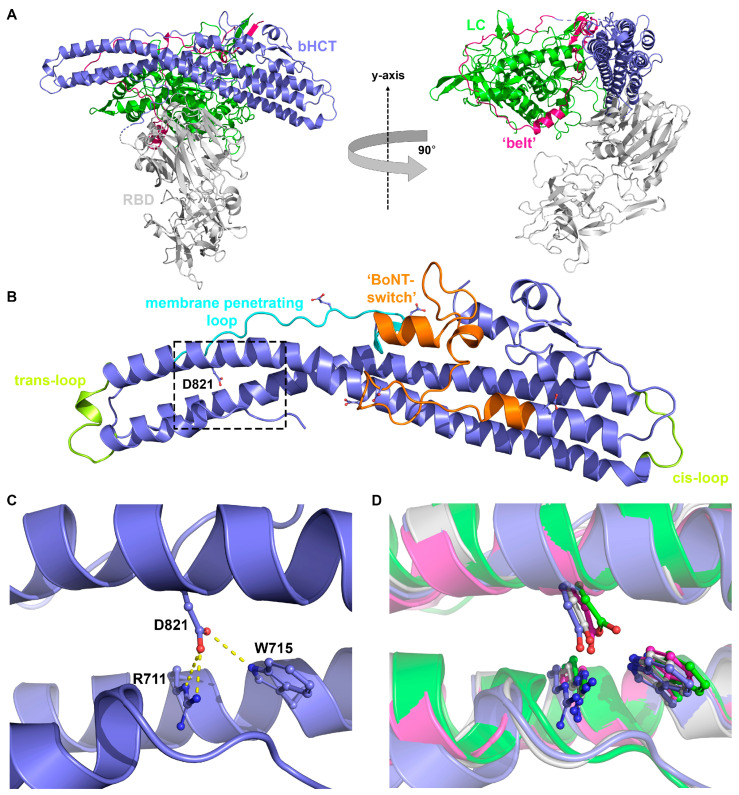
(**A**) Overall structure of TeNT (PDB: 5N0B) with the catalytic domain (LC, green), the belt region (pink), the beltless translocation domain (bHCT, blue) and the receptor-binding domain (RBD, silver). (**B**) Enlarged view of the beltless translocation domain highlighting secondary structures implicated in LC translocation: “BoNT-switch” (orange); membrane-penetrating loop (cyan); *trans*- and *cis*-loops (yellow). In addition, the six D/E residues in TeNT conserved across the CNT family are shown as sticks (atomic color). The boxed region containing D821 is expanded in panel C. (**C**) Details of the interface between the two central α-helices (blue): residues Arg711-Trp715-Asp821 involved in the interface are shown as sticks (atomic color). Hydrogen bonds are indicated by yellow dashed lines. (**D**) Superposition of the cartoon representations of the crystal structures of BoNT/A (PDB: 3BTA, green), BoNT/B (PDB: 1EPW, silver), BoNT/E (PDB: 3FFZ, pink) and TeNT (PDB: 5N0B, blue) showing the structural conservation of the Arg-Trp-Asp triad.

Tetanus has been a major infectious disease throughout human history that has now been largely eliminated from developed countries through effective vaccination programs [1]. In contrast to tetanus, botulism is a rare disease in humans for which there is no approved vaccine to prevent disease. Tetanus (TeNT) and botulinum (BoNT) neurotoxins, the causative agents of tetanus and botulism, respectively, are the most potent toxic molecules known to humankind, with an estimated median lethal dose (LD_50_) of less than 1 ng/kg of toxin per body weight [2,3]. While only a single type of TeNT is known, BoNTs are classified into eight serotypes (BoNT/A–G, BoNT/X) [4,5,6]. Collectively, TeNT and BoNTs belong to the same toxin family, known as clostridial neurotoxins (CNTs). Like all CNTs, mature TeNT is expressed as a polypeptide of ~150 kDa, which is post-translationally cleaved by one or more bacterial proteases into an active di-chain form linked by an essential interchain disulfide bond (Figure 1A) [7,8,9]. The N-terminal light chain (LC, Figure 1A, green) is the catalytic domain, a 50 kDa zinc protease that specifically cleaves synaptobrevin (types 1 and 2), also known as vesicle-associated membrane protein (VAMP types 1 and 2) [10]. The heavy chain (HC) consists of two ~50 kDa domains, each with distinct functions. The N-terminal translocation domain (HCT, Figure 1A, blue) facilitates the delivery of the LC into the cytosol [11], and the C-terminal receptor-binding domain (RBD Figure 1A, silver) is responsible for neuronal binding [12]. Upon translocation across the endosomal membrane, the interchain disulfide bond is reduced by the thioredoxin reductase–thioredoxin redox system [13], releasing the LC into the cytosol where it can cleave VAMP 2 and prevent synaptic vesicle exocytosis.

The N-terminus of HCT contains a long and unstructured region known as the “belt” (Figure 1A, pink), which wraps around the LC in a manner reminiscent of the BoNT/A substrate SNAP-25 [14]. This arrangement is conserved by the belts of TeNT [9], BoNT/B [15] and BoNT/E [16]. The precise role of the belt in the holotoxin is unclear, as it appears necessary for the translocation of the LC to occur [17] yet is dispensable for the formation of cation-conducting channels in lipid bilayers [18]. Thus, further studies of the belt will be needed to determine whether it plays a physiological role during LC translocation beyond simply anchoring the LC to the remainder of the HC. Immediately following the belt is a series of largely unstructured loops that lead into the so-called “BoNT-switch” domain (Figure 1B, orange). The BoNT-switch (BoNT/A residues 620–666, PDB: 6 MHJ) undergoes a transition from an α-helix bundle to a β-strand, which is proposed to favor the interaction of the translocation domain with the membrane [19]. While direct experimental evidence is currently lacking, it is assumed that the corresponding region in the remaining CNTs would undergo a similar structural transition at low pH. Downstream of the switch region is the “membrane-penetrating loop” (Figure 1B, cyan), which contains a short motif (residues 669–691 in TeNT) capable of forming cation-conducting channels in lipid bilayers [20]. However, while Electron Paramagnetic Resonance analyses of spin-labeled BoNT/A suggest that the membrane-penetrating loop undergoes significant conformational change as the toxin associates with the membrane, it does not support the conclusion that the region enters into the core of the bilayer [21].

The most salient feature of HCT is a pair of ~11 nm α-helices, anti-parallel and amphipathic, that twist around each other like a coiled coil. The two central α-helices are surrounded by four smaller α-helices. A recent study described the *trans*-loop region (Figure 1B, yellow) as linking the tips of the two central α-helices at the end of HCT that is physically distanced from the interchain disulfide connecting the LC to the HC, with the *cis*-loop (Figure 1B, yellow) described as the region located closest to the LC-HC interchain disulfide [22]. Directed mutagenesis identified a role for TeNT K768, located within the *cis*-loop, in LC translocation that did not interfere with other toxin functions including cell binding, intracellular trafficking and membrane pore formation. However, while the K768 residue in TeNT appears essential for proper translocation, the homologous residue in BoNT/A does not affect toxin cell entry [23]. Thus, while there are a growing number of structural and biochemical insights into the function of the HCT domain, many questions remain.

Recently, we used various spectroscopic techniques to demonstrate that a beltless variant of the TeNT translocation domain (bHCT) inserts into anionic membranes only under conditions of low pH and that insertion was accompanied by membrane-induced refolding [24,25]. While membrane-induced refolding and insertion were highly dependent on acidic pH, the specific amino acids that have to be protonated to induce the structural rearrangements are essentially unknown. In the case of the prototypic diphtheria toxin, histidines have been proposed to function as such “molecular switches” both in solution and at the membrane [26,27], with a relatively minor role played by acidic residues [28]. By comparison, the bHCT of TeNT is devoid of histidine residues but possesses a number of aspartate and glutamate residues that are highly conserved across the CNT family. Thus, we postulated that the protonation of one or more of these conserved residues is responsible for the pH-triggered membrane insertion of HCT. Using full-length and truncated forms of tetanus neurotoxin, in conjunction with biochemical and cellular assays, we provide evidence that a strictly conserved Asp-Arg-Trp triad within the translocation domain plays a critical role in sensing and responding to environmental pH.

## 2. Results

**Figure 2:**
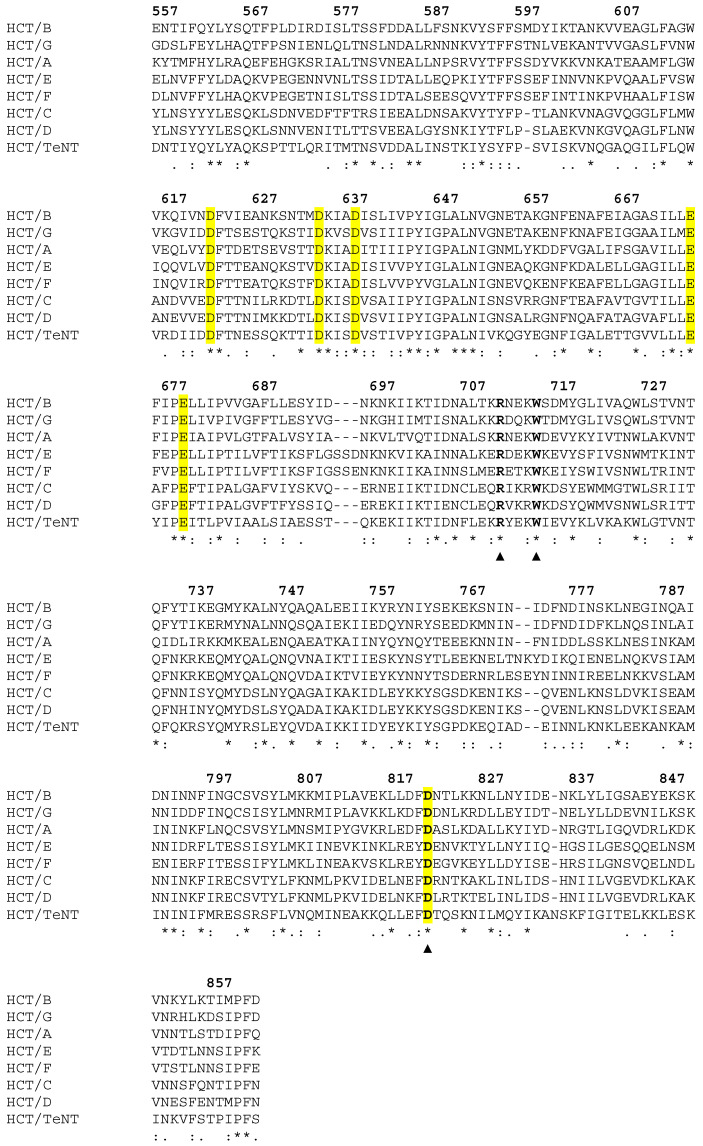
Multiple sequence alignment of the beltless translocation domain of botulinum neurotoxins A–G and tetanus neurotoxin using CLUSTALW. An * (asterisk) indicates positions which have a single, fully conserved residue. Conserved acidic residues (D and E) are highlighted in yellow. The Arg711-Trp715-Asp821 triad is highlighted with ▲ symbols. Numbering refers to the sequence of tetanus neurotoxin. Here, we focus on aspartate 821 as one potential “molecular switch”, involved in triggering the conformational changes in HCT necessary for insertion into the membrane bilayer. Our rationale for choosing to focus on D821 is as follows: (i) due to its central position at the interface between the two extended amphipathic α-helices of HCT (Figure 1B,C), the protonation of D821 could very likely destabilize intramolecular contacts with arginine 711 and tryptophan 715 upon acidification; (ii) D821 and the interacting residues R711 and W715 are strictly conserved across the CNT family (Figure 1D and Figure 2); and (iii) D821 is located within a region of the central α-helix previously shown in BoNT/A to be protease-resistant in the context of proteoliposomes [21], suggesting insertion into or close association with the membrane bilayer.

While histidines are implicated in triggering the translocation process of several AB toxins [29,30,31], only a single non-conserved histidine is found within the tetanus toxin translocation domain. In contrast, several conserved acidic residues (D, E) have been identified within the bHCT domain of the clostridial neurotoxin family which may function to regulate the translocation process (Figure 2).

Initially, a subset of structurally diverse amino acids was selected as substitutes for D821, with the effects on protein expression and purification determined. Asparagine was selected to replace D821 (D821N) as it is structurally most similar to aspartic acid and thus might be able to interact with neighboring residues at the interface, similarly to aspartic acid. In contrast, alanine at position 821 (D821A) was predicted to abrogate any interactions of D821 with neighboring residues, which would provide information about the structural significance of aspartic acid at the position. Arginine (D821R), with its large sidechain and positive charge, was expected, at first glance, to destabilize intramolecular interactions, leading to a disruption of the tertiary structure of HCT. Of the three substitutions tested, only HCT ^D821N^ could be expressed and purified with similar characteristics to the wildtype protein. Thus, HCT ^D821A^ and HCT ^D821R^ were not analyzed further. A similar analysis was performed for R711, demonstrating that substitutions R711Q and R711K were well tolerated. As previously reported [25], the substitution of W715 was particularly problematic, with replacements to Phe, Tyr, Ala, Met and Ile all failing to yield soluble protein. However, HCT ^W715C^ could be expressed and purified with characteristics indistinguishable from the wildtype protein. Moreover, the W715C substitution did not appear to affect function when introduced into the mature holotoxin as judged by the intoxication of primary neurons (Figure S1).

### 2.1. Effect of Mutations on Liposome Permeabilization

**Figure 3:**
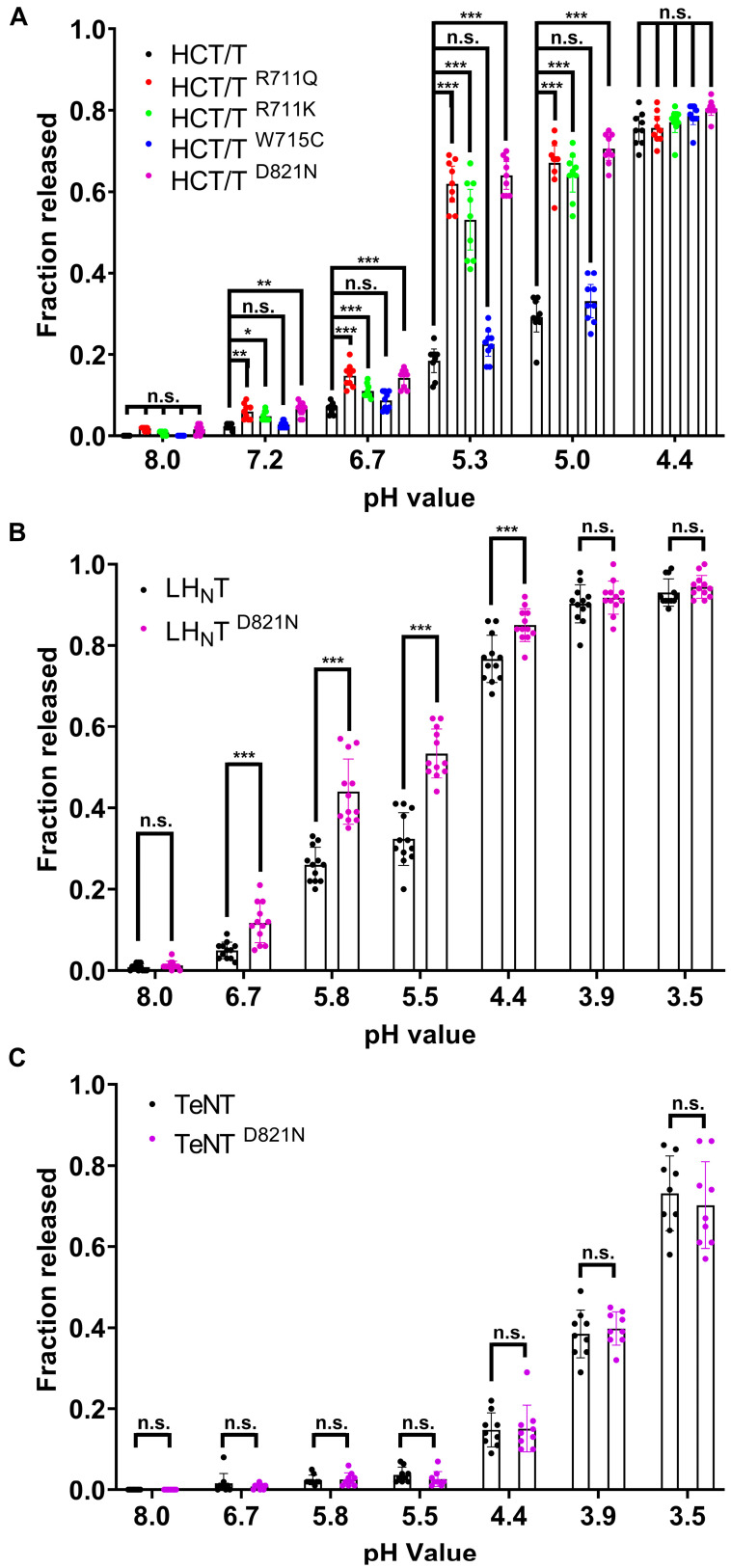
Leakage of ANTS/DPX from liposomes in response to wildtype and mutated tetanus toxin proteins. The leakage of ANTS/DPX probes from 65POPC:25POPS:10Chol liposomes was measured after 15 min exposure to wildtype or mutated HCT (**A**), LH_N_T (**B**) and full-length toxins (**C**) at the indicated pH. The fluorescence (at 530 nm) of undisturbed LUVs was set to 0% and that of vesicles disrupted by Triton X-100 was set to 100%. Results are expressed as mean values ± S.D. (error bars) for a minimum of 9 independent liposome preparations. *, *p* < 0.05; **, *p* < 0.001; ***, *p* < 0.0001; n.s., not significant. Two-way analysis of variance with Tukey’s Multiple Comparison Test.

To assess the effect of mutations on the low-pH-triggered translocation process, we first used a well-established liposomal leakage assay [32]. The addition of wildtype HCT to liposomes containing anionic lipids resulted in the pH-dependent release of ANTS (8-Aminonaphthalene-1,3,6-Trisulfonic Acid) dye, which did not reach a maximum until pH 4.4 (Figure 3A). The mutation of W715 to Cys (W715C) did not appear to alter the pH dependence of liposome permeabilization. By comparison, the mutation of D821 (D821N) and R711 (R711Q or R711K) increased the estimated midpoint of release by approximately 0.5 pH units (Figure 3A). Next, we tested whether the change in the pH response of HCT could be observed in the presence of one or both of the other toxin domains. A TeNT variant containing the LC and HCT domains, hereafter referred to as LH_N_T, caused a similar level of dye release to the HCT domain, albeit at a slightly lower final pH (Figure 3B). We posit that lower pH is necessary to facilitate the unfolding of both the LC and HCT domains. As was observed with HCT, the mutation of D821 (LH_N_T ^D821N^) shifted the estimated midpoint of release relative to the wildtype protein (Figure 3B). Unexpectedly, the mutation of Arg711 to either Gln or Lys in the context of the LH_N_T fragment drastically reduced the expression of soluble protein and thus was not analyzed further. Finally, we compared the ability of full-length TeNT to permeabilize liposomes to that of TeNT ^D821N^. The overall extent of liposome permeabilization by TeNT was reduced and occurred at a lower pH value as compared to LH_N_T or HCT. However, no difference in liposome permeabilization was observed between TeNT and TeNT ^D821N^ (Figure 3C).

### 2.2. Effect of Mutations on Thermal Stability and Global Protein Structure

**Figure 4:**
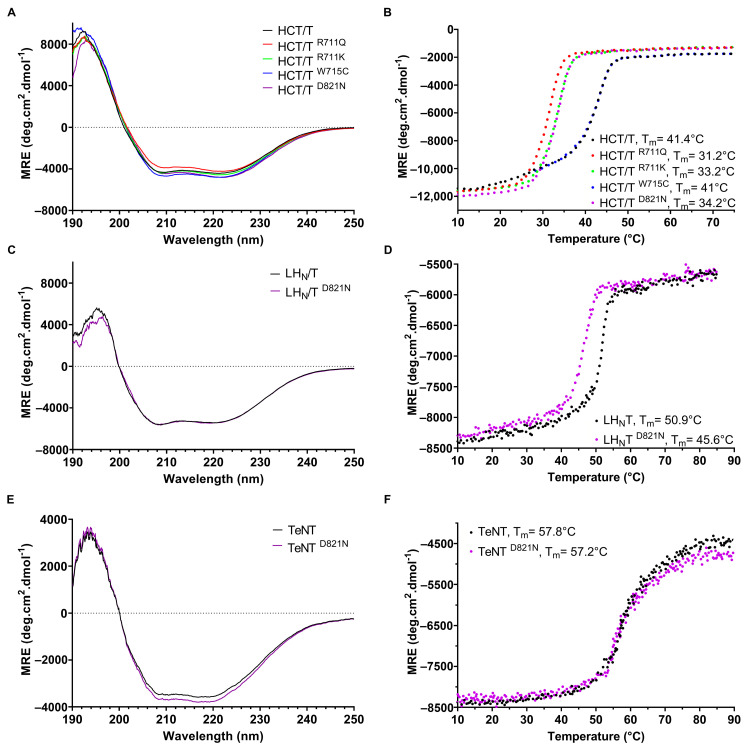
Tetanus toxin thermal denaturation monitored by CD spectroscopy. Average of four far-UV CD spectra of wildtype or mutated HCT (**A**), LH_N_T (**C**) and TeNT (**E**) recorded at 24 °C. In all cases, final protein concentration was 2.5 µM diluted in phosphate buffer at pH 8.0. The CD data are presented as mean residue molar ellipticity ([θ] MRW). Changes in ellipticity at 222 nm with the increase in temperature for wildtype or mutated HCT (**B**), LH_N_T (**D**) and TeNT (**F**). Data were fit to a two-state equilibrium unfolding model to estimate the thermal denaturation temperature (T_m_) of each sample. Data are representative of measurements made with at least 2 independent protein preparations.

To determine whether mutations affect the thermal stability and overall global protein structure, circular dichroism (CD) measurements were obtained. At pH 8.0, the far-UV CD spectra of wildtype HCT, LH_N_T and TeNT were characteristic of proteins with substantial α-helical content (Figure 4A,C,E), consistent with the known crystal structure of the toxin (PDB: 5N0B). The spectra of the mutated proteins were similar to those of their wildtype equivalents, suggesting that the indicated mutations do not noticeably affect the overall secondary structure. The thermal stability of wildtype and variant proteins was assessed by observing changes in CD intensity at 222 nm from 10 to 90 °C. HCT and HCT ^W715C^ were both denatured in a single-step process with melting temperatures of 41.4 °C and 41.0 °C, respectively. By contrast, HCT containing mutations of either R711 or D821 exhibited single transitions all with melting temperatures approximately 7–10 °C lower than the wildtype protein (Figure 4B). A similar pattern was observed for the LH_N_T fragment, with LH_N_T ^D821N^ displaying a melting temperature approximately 5 °C lower than LH_N_T (Figure 4D). In contrast, no significant difference in the thermal stability of the full-length TeNT and TeNT ^D821N^ proteins was observed (Figure 4F).

### 2.3. Mutations of Arg711 and Asp821 Alter the pH Transitions of 1-Anilinonaphthalene 8-Sulfonate (ANS) Binding

**Figure 5:**
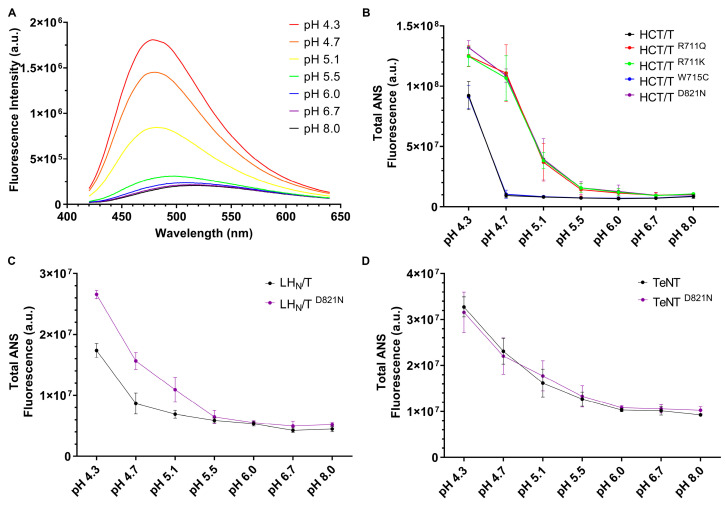
ANS binding to tetanus toxin. (**A**) ANS fluorescence spectra in the presence of wildtype HCT at the indicated pH values. Protein concentration was 2.5 μM, and concentration of ANS was 100 µM. Each spectrum is the average of three individual scans. Total ANS fluorescence was determined by calculating the area enclosed by the emission spectrum curve and plotted at the pH value for wildtype or mutated HCT (**B**), LH_N_T (**C**) and TeNT (**D**). Results are expressed as arithmetic mean ± SD from 3 independent determinations.

Both the emission wavelength maximum (λ_max_) and quantum yield of the ANS dye are dependent upon the polarity of the surrounding medium: an increase in quantum yield and a blue shift indicate the binding of the dye to hydrophobic regions of proteins [33]. When ANS is incubated in the presence of HCT at pH 4.3, the fluorescence intensity increases approximately seven-fold and the ANS λ_max_ is shifted from 510 to 470 nm relative to what was observed in the presence of HCT at pH 8.0 (Figure 5A). An estimation of the total ANS fluorescence, as determined by calculating the area under the spectra, reveals a transition in binding occurring close to pH 4.7 (Figure 5B). Consistent with the liposome permeabilization assays (Figure 3), mutations of either R711 or D821 revealed that the transition in ANS binding occurred at a higher pH value (~5.1, Figure 5B) relative to the wildtype. A similar transition in ANS binding was observed for LH_N_T and LH_N_T ^D821N^ relative to the HCT proteins (Figure 5C). Consistent with prior observations, no differences in ANS binding could be observed between TeNT and TeNT ^D821N^ (Figure 5D).

### 2.4. TeNT and LH_N_T Mutants Display Differential Activity for VAMP 2

**Figure 6:**
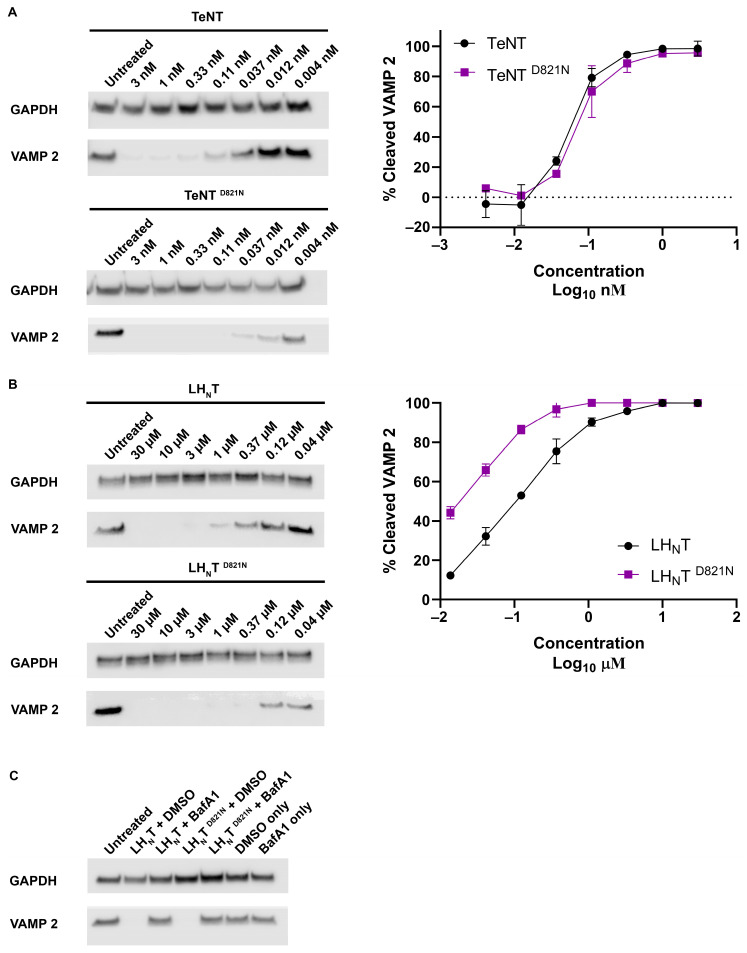
Cleavage of VAMP 2 by wildtype and mutant tetanus toxins. Cells were incubated in the presence of the indicated doses of either wildtype or mutant D821N tetanus toxins (**A**) or LH_N_Ts (**B**). After 16 h of incubation, cells were lysed. Lysates were subjected to Western blotting with antibodies against VAMP 2 and GAPDH (loading control). Images are representative of three independent experiments. Quantification was performed using ImageJ version 1.54p. The relative cleavage of VAMP 2 was calculated by normalizing the quantification values of VAMP 2 band intensity to the total GAPDH signal. (**C**) Cells were incubated for 30 min with either 400 nM bafilomycin A1 (BafA1) or solvent (DMSO) prior to the addition of 30 µM LH_N_T or LH_N_T ^D821N^. VAMP 2 cleavage was visualized and quantified as described above.

We first tested the overall functionality of recombinant wildtype and mutant TeNT in rat cortical neurons, which are highly sensitive to CNTs and provide a rapid and reliable method to assay the cleavage of VAMP 2 by Western blot. After 16 h of incubation, TeNT and TeNT ^D821N^ cleaved VAMP 2 with similar efficiency (Figure 6A).

The extent of cleavage was within 10-fold of that achieved with “natural TeNT” purified from Clostridium tetani, implying that production in Bacillus megaterium generates a fully biologically active molecule. Prior studies indicated that the LH_N_T fragment of both botulinum and tetanus toxins can enter neurons without the aid of the RBD [17,24]. Given our earlier observations (Figure 2, Figure 3, Figure 4 and Figure 5), we posited that the presence of the RBD in the full-length toxins may be suppressing the effect of the D821N mutation. We therefore decided to repeat the analysis using LH_N_T. Consistent with prior observations, LH_N_T and LH_N_T ^D821N^ both cleaved VAMP 2, albeit at much higher concentrations relative to full-length toxins. However, contrary to what was observed for the full-length proteins, the cleavage of VAMP 2 by LH_N_T ^D821N^ was consistently observed at concentrations at least three-fold lower than for the wildtype (Figure 6B). To further validate the use of LH_N_T as a tool to study translocation, the requirement for passage through an acidified compartment was investigated. In agreement with previous studies, the activity of LH_N_T was inhibited by the vacuolar proton pump inhibitor bafilomycin A1 (Figure 6C) [24]. Similarly, the activity of LH_N_T ^D821N^ was also dependent on exposure to an acidified environment (Figure 6C), suggesting that VAMP 2 cleavage was not occurring post lysis of the neurons.

### 2.5. Mutation of Glutamate 675 Enhances Membrane Permeabilization Activity of bHCT

**Figure 7:**
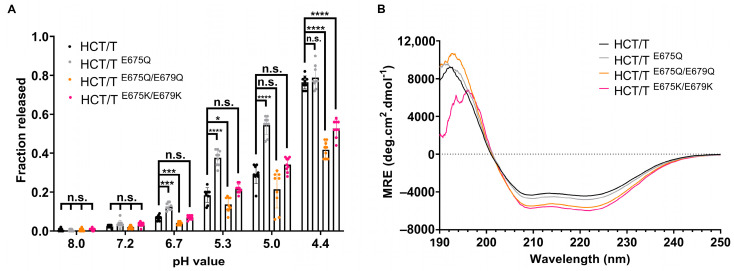
Leakage of ANTS/DPX from liposomes in response to wildtype and mutated tetanus toxin proteins. (**A**) The leakage of ANTS/DPX probes from 65POPC:25POPS:10Chol liposomes was measured after 15 min exposure to wildtype or mutated bHCT at the indicated pH. The fluorescence (at 530 nm) of undisturbed liposomes was set to 0% and that of vesicles disrupted by Triton X-100 was set to 100%. Results are expressed as mean values ± S.D. (error bars) for a minimum of 9 independent liposome preparations. *, *p* < 0.05; ***, *p* < 0.001; ****, *p* < 0.0001; n.s., not significant. Two-way analysis of variance with Tukey’s Multiple Comparison Test. (**B**) Average of four far-UV CD spectra of wildtype or mutated bHCT recorded at 24 °C. Final protein concentration was 2.5 µM diluted in phosphate buffer at pH 8.0. The CD data are presented as mean residue molar ellipticity ([θ] MRW).

Previously, it was shown that a botulinum toxin B triple mutant (E48Q, E653Q, D877) showed increased neurotoxicity due to faster cytosolic delivery of the LC, with translocation occurring at a less acidic pH [34]. Sequence- and structure-based alignments indicate that E653 of botulinum toxin B is highly conserved across the CNT family and is located at the junction of the BoNT-switch region and the membrane-penetrating loop. We therefore tested whether the mutation of the corresponding residue in the bHCT of tetanus toxin (E675) could modulate the interaction of the domain with membranes. Figure 7A demonstrates that HCT ^E675Q^, similarly to HCT ^D821N^, permeabilized liposomes under less acidic conditions as compared to the wildtype protein. Interestingly, the double mutation of E675 and E679 (also strictly conserved in the CNT family) to either glutamine or lysine impaired the ability of the mutants to permeabilize liposomes without causing significant alterations to the secondary structure of the bHCT (Figure 7B).

## 3. Discussion

While the overall mechanism by which clostridial neurotoxins (CNTs; tetanus and botulinum neurotoxins) are able to cause paralytic diseases is well characterized, there remains a large gap in our understanding of how the translocation domain (HCT) delivers the light chain (LC) to the cytosol. Recently, we showed that the refolding–membrane insertion of a beltless HCT (bHCT) occurs at the membrane interface—a process that is triggered by low pH [25]. Working on the assumption that the protonation of one or more key titratable residues triggers the refolding–membrane insertion of bHCT, we identified a total of four aspartate and two glutamate residues that were strictly conserved across the clostridial neurotoxin family (Figure 2). The available crystal structures of tetanus neurotoxin (TeNT; PDBs: 5N0B and 7BY5) suggest that two of the six residues form polar contacts with adjacent residues in bHCT. D636, located within the BoNT-switch region, forms hydrogen bonds with R802 and S803. While we speculate that D636 could be involved in sensing low pH in bHCT, it is important to note that neither R802 nor S803 is strictly conserved across the CNT family (Figure 2). The second residue, D821, resides in the interface between the two central α-helices and forms polar contacts with residues located on the adjacent central α-helix of bHCT: a side-on interaction with the guanidinium group of R711 and an additional hydrogen bond with the indole N-H group of W715 (Figure 1C). Importantly, the R711-W715-D821 interactions observed in TeNT appear to be strictly conserved in all members of the CNT family (Figure 1D and Figure 2). Thus, we posited that the protonation of D821 could destabilize interactions between the two extended α-helices of bHCT as a key step in refolding–membrane insertion. To test this possibility, site-directed mutagenesis was employed to generate D821N, R711K/Q and W715C mutations.

In the context of bHCT, mutations D821N and R711K/Q (i) permeabilized large unilamellar vesicles (LUVs) under less acidic conditions as compared to the wildtype protein (Figure 3); (ii) displayed reduced thermal stability, as assessed by circular dichroism spectroscopy (Figure 4); and (iii) showed an enhancement of ANS fluorescence under less acidic conditions (Figure 5). A similar pattern was also observed for the D821N mutation in the context of the LH_N_T fragment (composed on the LC and HCT domains only), albeit with LUV permeabilization occurring at lower pH values relative to the HCT. This is perhaps unsurprising given that the LC domain also has to undergo pH-triggered unfolding prior to translocation across the membrane. Finally, we demonstrated that LH_N_T ^D821N^ was more efficient at cleaving VAMP 2, as compared to wildtype LH_N_T, possibly due to faster kinetics of toxin entry (Figure 6). These findings are consistent with the proposition that the protonation of D821 helps disrupt the interaction between the two central α-helices of HCT to promote refolding–membrane insertion.

While the D821N and R711K/Q mutations in the context of the HCT and LH_N_T fragments behaved as predicted, the observed effects on permeabilization, thermal stability and ANS binding appear to be negated by the presence of the receptor-binding domain (RBD, full-length TeNT, Figure 3, Figure 4, Figure 5 and Figure 6). Previous studies demonstrated that while the RBD of botulinum toxin A is not necessary for in vitro channel formation or LC translocation, it does appear to dictate the pH threshold of HCT insertion into the membrane [17,18,35]. In a separate study, it was shown that a botulinum toxin B triple mutant (E48Q of the LC, E653Q of the HCT and D877 of the RBD) showed increased neurotoxicity due to faster cytosolic delivery of the LC, with translocation occurring at a less acidic pH [34]. Importantly, however, the protonation of D877 within the RBD appeared indispensable for subsequent steps, as the mutation of E48 and E653 alone or together did not show effects on the activity of BoNT/B. While the precise mechanism by which the RBD seemingly overrides mutations in HCT is currently unclear, we speculate that the RBD functions in part to ensure that refolding–membrane insertion occurs at the appropriate location within the intoxicated neuron.

Interestingly, E653 of BoNT/B is strictly conserved across the CNT family and is located at the junction of the BoNT-switch region and the membrane-penetrating loop. While the mutation of E653 in the BoNT/B triple mutant resulted in increased toxicity, the study was not able to resolve the molecular basis of this effect [34]. An analysis of the corresponding residue in the bHCT of tetanus toxin (E675) demonstrates that HCT ^E675Q^ permeabilized LUVs under less acidic conditions as compared to the wildtype protein (Figure 7A). Far-UV CD measurements suggest that the E675Q mutation does not cause gross changes in secondary structure, at least in the absence of membranes (Figure 7B). The simplest explanation is that the protonation of E675 removes insertion blocking charge from the junction of the BoNT-switch region and the membrane-penetrating loop. This would allow the conserved β2/β3 loop of the BoNT-switch region to interact with the membrane bilayer, bringing the central α-helices into proximity with the membrane. The interaction of HCT with membrane lipids, in conjunction with the protonation of D821N, could then facilitate the structural refolding necessary to promote LC translocation. In conclusion, we have identified a key interaction between R711 and D821 that is involved in both stabilizing the HCT domain and promoting the pH-triggered refolding–membrane insertion. Furthermore, our data support a model in which a cascade of titratable residues, located across multiple domains of the holotoxin, is required to facilitate the translocation of LC into the cytosol.

## 4. Materials and Methods

Molecular biology-grade reagents were purchased from either Thermo Fisher Scientific (Waltham, MA, USA) or Millipore Sigma (Burlington, MA, USA) unless otherwise stated. Cholesterol was purchased from Matreya, LLC (State College, PA, USA). A mini extruder unit and the following phospholipids were purchased from Avanti Polar Lipids (Alabaster, AL, USA): 1-palmitoyl-2-oleoyl-sn-glycero-3-phosphocholine (POPC) and 1-palmitoyl-2-oleoyl-phosphatidylserine (POPS).

### 4.1. Construction of Toxin Expression Vectors

*Bacillus megaterium* optimized DNA encoding tetanus toxin (TeNT) appended to a Strep Tag II epitope at the C-terminus was synthesized by Genscript (Piscataway, NJ, USA). TeNT DNA was then subcloned into the *B. megaterium* expression vector pHis1522 (MoBiTec GmbH, Goettingen, Germany), such that that the resulting protein contained an N-terminal hexahistidine. DNA encoding the light-chain and heavy-chain translocation domains (LH_N_T, residues 1–868) with a C-terminal extension encoding a Strep Tag II epitope was amplified by polymerase chain reaction and cloned into pHis1522 such that the resulting protein contained an N-terminal hexahistidine tag. The beltless translocation domain (HCT, residues 555–868) was cloned and expressed as described previously [25]. In all cases, DNA fragment insertion was confirmed by automated Sanger sequencing (MU Genomics Technology Core, Columbia, MO, USA).

### 4.2. Site-Directed Mutagenesis of Toxin Constructs

Point mutations were generated using the QuikChange Lightning site-directed mutagenesis kit (Agilent Technologies, Santa Clara, CA, USA). Mutagenesis was confirmed by automated Sanger sequencing (MU Genomics Technology Core, Columbia, MO, USA). Primers used for the introduction of point mutations are shown in Table S1.

### 4.3. Protein Expression

*Bacillus megaterium* cells transformed with pHis1522 plasmids were cultured overnight on LB agar with 10 μg/mL tetracycline at 37 °C. Transformants were subsequently cultured in 3 mL LB broth with 10 μg/mL tetracycline and then stored at −80 °C in 30% (*v*/*v*) glycerol. For protein expression, *B. megaterium* containing pHis1522 expression plasmids were grown for ~18 h on LB agar plates with 10 μg/mL tetracycline at 37 °C. Subsequently, a single colony was introduced into 100 mL LB broth with 10 μg/mL tetracycline and grown overnight. The following day, 10 mL aliquots of the culture were introduced into 10 × 400 mL of LB broth with 10 μg/mL tetracycline. Cultures were grown for ~6 h at 30 °C until an optical density at 600 nm (OD_600_) of 0.6 was reached. Xylose was then added to a final concentration of 0.5% *w*/*v*, and culture continued for an additional 18 h.

### 4.4. Protein Purification

Cells were collected by centrifugation (7500× *g*, 15 min, 4 °C) and brought up in ice-cold Ni^2+^-NTA binding buffer (BB, 20 mM Tris, 500 mM NaCl, 5 mM imidazole, pH 8) supplemented with protease inhibitor cocktail (1:100) and universal nuclease (1:5000). Cells were lysed in a French press and clarified by centrifugation and filtration. The filtered lysate was applied to a Ni^2+^-NTA (nitrilotriacetic acid) column (Qiagen, LLC, Germantown, MD, USA), washed with BB with 20 mM imidazole and bound proteins eluted with elution buffer (20 mM Tris, 500 mM NaCl, 250 mM imidazole, pH 8). Peak fractions were pooled, diluted with StrepTactin binding buffer (SBB, 100 mM Tris, 150 mM NaCl, 10% *w*/*v* glycerol, pH 8) and applied three times to a StrepTactin Sepharose column (IBA Lifesciences, Göttingen, Germany). The column was then washed with 45 mL SBB and eluted with StrepTactin elution buffer (100 mM Tris, 150 mM NaCl, 10% *w*/*v* glycerol, 10 mM desthiobiotin, pH 8). Peak fractions were pooled and concentrated using Amicon devices to a minimum of 5 mg/mL, aliquoted and stored at −80 °C until use. To estimate protein purity, samples (5 μg) were separated by SDS-PAGE and visualized by staining with Colloidal Coomassie blue. All proteins used in this study were estimated to be at least 90% pure (Figure S2A).

### 4.5. Trypsinization of TeNT Toxin and Fragments

Tetanus toxin and LH_N_T were incubated with trypsin at a 1:1000 (*w*/*w*) trypsin/protein ratio in 20 mM potassium phosphate and 50 mM NaCl (pH 8.0) for 90 min at 37 °C, after which a 5-fold molar excess (with respect to trypsin) of soybean trypsin inhibitor was added. Control reactions—subjected to SDS-PAGE (with or without beta-mercaptoethanol) and stained with Coomassie blue—demonstrated that >90% of the single-chain protein was converted to the di-chain form which remained associated through a disulfide bond (Figure S2B).

### 4.6. Liposome Permeabilization Assay

Liposomes were prepared by mixing lipids dissolved in chloroform in the indicated molar ratios. Solvent was evaporated under a gentle stream of nitrogen followed by placing samples under high vacuum overnight. Tris buffer (20 mM Tris–HCl, pH 8.0) containing 9 mM ANTS (8-Aminonaphthalene-1,3,6-Trisulfonic Acid) and 20 mM DPX (*p*-Xylene-Bis-Pyridinium Bromide), was then added to bring lipids to a 30 mM final concentration. These suspensions were placed in a water bath at 60 °C and vortexed every 20 min for 3 h to hydrate the lipid film. The hydrated lipid was then subjected to 10 cycles of freeze–thawing followed by extrusion through two 0.1 μm Nucleopore polycarbonate membranes for 25 passes. To separate unencapsulated molecules from LUVs, samples were loaded onto a column containing 12 mL Sephadex G50 resin pre-equilibrated in 20 mM Tris–HCl and 50 mM NaCl, pH 8.0. The vesicle stocks were stored at 4 °C until use for a maximum of two days.

Liposomes were diluted to a final concentration of 600 µM in 20 mM Tris–HCl and 50 mM NaCl, pH 8.0, with constant stirring and allowed to equilibrate for 5 min. Tetanus toxin or fragments thereof were then added to the solution to a final concentration of 50 nM (LH_N_T or HCT, protein/lipid = 1:12,000) or 250 nM (TeNT, protein/lipid = 1:2400), and incubation continued for a further 5 min. After equilibration, a defined amount of 2.5 M acetic acid was added to rapidly bring the sample to the indicated pH and the change in fluorescence recorded for a 15 min period. Triton X-100 was then added to a final concentration of 0.2% *v*/*v* to fully lyse the LUVs, and fluorescence corresponding to 100% ANTS was determined. Fractional leakage was then calculated as follows: (F_sample_ − F_initial_)/(F_lysed_ − F_initial_) × 100%. An Edinburgh Instruments FS5 spectrofluorometer (Livingston, United Kingdom) was used to record ANTS fluorescence emission at 530 nm upon excitation of the sample at 350 nm, with the excitation and emission bandwidths set to 1 nm and 5 nm, respectively.

### 4.7. ANS Binding Assay

Tetanus toxin or fragments thereof were diluted in buffer at various pH values (20 mM Tris–HCl, 50 mM NaCl, pH 8.0; 20 mM Tris–HCl, 50 mM NaCl, pH 6.7; 20 mM Mes-OH, 50 mM NaCl, pH 6.0; 20 mM Mes-OH, 50 mM NaCl, pH 5.5; 20 mM Mes-OH, 50 mM NaCl, pH 5.1; 20 mM sodium acetate, 50 mM NaCl, pH 4.7; 20 mM sodium acetate, 50 mM NaCl, pH 4.3) to give a final concentration of 2.5 µM in the presence of 100 µM 1-anilinonaphthalene 8-sulfonate (ANS) and equilibrated for 20 min at room temperature. Samples containing ANS in the absence of toxin fragments were used to correct for effects of pH alone. An Edinburgh Instruments FS5 spectrofluorometer was used to record emission spectra between 425 and 640 nm upon excitation of the sample at 370 nm, with the excitation and emission bandwidths set to 1 nm and 5 nm, respectively. The total emitted fluorescence was estimated by calculating the area enclosed by the emission spectrum curve using OriginPro software version 2019 (9.6.0.172) (OriginLab Corporation, Northampton, MA, USA).

### 4.8. Circular Dichroism

Circular dichroism (CD) measurements were performed using a Jasco J-1500 CD spectrometer (Tokyo, Japan) equipped with a Jasco PTC-517 Peltier cell holder. The protein samples were diluted to 2.5 µM in 10 mM potassium phosphate buffer (pH 8.0) in a quartz cuvette with a path length of 0.1 cm. Data were recorded between 250 nm and 190 nm using a 1 nm bandwidth and a 4 **s** response for full circular dichroism spectra. Protein spectra were corrected with signal subtraction by protein-free phosphate buffer. Thermal denaturation was measured at 222 nm with a heating rate of 1 °C/min, raising the temperature interval of 1 °C from 10 to 90 °C. Data were fit to a two-state equilibrium unfolding model in OriginPro to determine the thermal denaturation temperature (T_m_) of each sample.

### 4.9. Culture of Primary Rat Cortical Neurons

Rat E18 cortical neurons (Brainbits, LLC, Springfield, IL, USA) were cultured on poly-d-lysine-coated glass coverslips in Neurobasal medium containing B-27 supplement for 14–21 days prior to use. Half of the culture medium was changed with fresh Neurobasal medium every 4 days starting on day 7.

### 4.10. Cellular Intoxication Assays

Cells were treated with trypsin-activated TeNT or TeNT variants at the indicated concentrations for 16 h at 37 °C. In some experiments, cells were pretreated for 30 min with either 400 nM bafilomycin A1 or solvent (DMSO) prior to the addition of the toxins. After incubation, cells were washed twice with cold PBS, lysed using radioimmunoprecipitation assay buffer at 4 °C for 20 min and clarified by centrifugation at 21,000× *g* for 20 min at 4 °C. Supernatants were combined with Laemmli sample buffer and subjected to SDS-PAGE and Western blot analysis using antibodies against VAMP 2 (clone 69.1, 1:5000, Synaptic Systems, Goettingen, Germany) and GAPDH (clone 14D10, 1:1000, Cell Signaling Technology, Danvers, MA, USA) and goat anti-mouse-IgG-HRP and goat anti-rabbit-IgG-HRP (1:100,000) secondary antibodies. The membranes were washed, incubated with SuperSignal Dura and visualized using a CCD imaging system (LI-COR Odyssey XF, LI-COR, Lincoln, NE, USA). Densitometric analysis of Western blots was performed using ImageJ version 1.54p [36].

## Data Availability

The original contributions presented in this study are included in this article and the Supplementary Materials. Further inquiries can be directed to the corresponding authors.

## Supplementary Materials

The following supporting information can be downloaded at https://www.mdpi.com/article/10.3390/toxins17060273/s1, Figure S1: Cleavage of VAMP 2 by wildtype and mutant tetanus toxins. Figure S2: Purification of TeNT and related proteins. Table S1. Primers used in this study.

## Abbreviations

The following abbreviations are used in this manuscript:

TeNT	Tetanus neurotoxin
BoNT	Botulinum neurotoxin
CNT	Clostridial neurotoxin
LC	Light chain
HC	Heavy chain
HCT	Heavy-chain translocation
RBD	Receptor-binding domain
LH_N_T	LC-HCT only
VAMP 2	Vesicle-associated membrane protein 2
CD	Circular dichroism
LUV	Large unilamellar vesicles
POPC	1-Palmitoyl-2-oleoyl-phosphatidylcholine
POPS	1-Palmitoyl-2-oleoyl-phosphatidylserine
ANTS	8-Aminonaphthalene-1,3,6-Trisulfonic Acid
DPX	*p*-Xylene-Bis-Pyridinium Bromide
ANS	1-Anilino-8-napthalene sulfonate
Ni^2+^-NTA	Ni^2+^–nitrilotriacetic acid
BB	Ni^2+^-NTA binding buffer
SBB	StrepTactin binding buffer
