# Supplementary material for: The Role of a Conserved Arg-Asp Pair in the Structure and Function of Tetanus Neurotoxin
Source: Toxins (Basel). 2025 May 30;17(6):273. doi: 10.3390/toxins17060273 (PMC12197568; doi:10.3390/toxins17060273)
Supplement: Supplementary file 1 [file toxins-17-00273-s001.zip › toxins-3573460-supplementary.pdf]

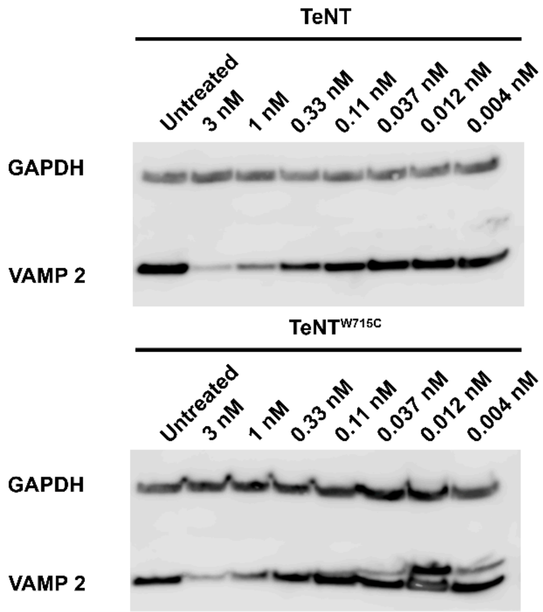

Figure 1. Cleavage of VAMP 2 by wildtype and W715C tetanus toxin. Cells were incubated in the presence of the indicated doses of either wildtype (**A**) or mutant W715C (**B**) tetanus toxins). After 16 h of incubation cells were lysed. Lysates were subjected to Western blotting with antibodies against VAMP 2 and GAPDH (loading control). Images are representative of at least two independent experiments.

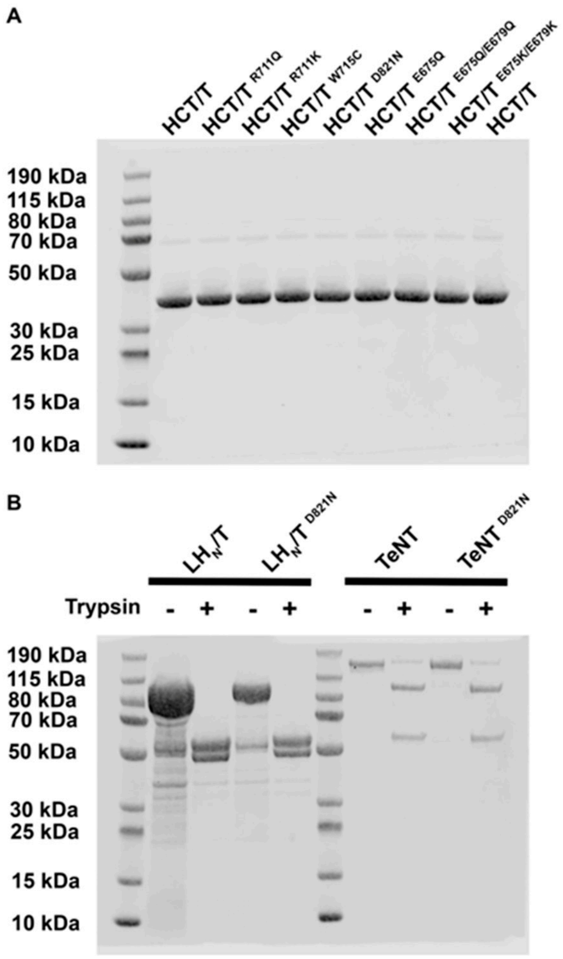

Figure 2. Purification of TeNT and related proteins. Individual plasmids encoding wildtype or mutated HCT were expressed in *E. coli* (**A**), while plasmids encoding wildtype or mutated LHNT (**B**) and wildtype or mutated TeNT were expressed in *B. megaterium*. Proteins were purified by affinity chromatography. Five micrograms of the indicated proteins were subjected to SDS-polyacrylamide gel electrophoresis. In the case of LHNT and TeNT proteins were incubated with or without trypsin for 90 min prior to electrophoresis. The gels were stained with Coomassie blue.

Table S1. Primers used in this study

**Name**	**Sequence 5′→3′**
D821N Forward	GAAAAAACAGCTGCTGGAATTTAATACCCAGAGCAAAAACATTC
D821N Reverse	GAATGTTTTTGCTCTGGGTATTAAATTCCAGCAGCTGTTTTTTC
R711Q Forward	CCATTGATAACTTTCTGGAAAAACAGTATGAAAAATGGATTGAAGTGTATAAACTGG
R711Q Reverse	CCAGTTTATACACTTCAATCCATTTTTCATACTGTTTTTCCAGAAAGTTATCAATGG
R711K Forward	CCATTGATAACTTTCTGGAAAAAAAGTATGAAAAATGGATTGAAGTGTATAAACTGG
R711K Reverse	CCAGTTTATACACTTCAATCCATTTTTCATACTTTTTTTCCAGAAAGTTATCAATGG
W715C Forward	CTGGAAAAACGTTATGAAAAATGCATTGAAGTGTATAAACTGGTGAAAGC
W715C Reverse	GCTTTCACCAGTTTATACACTTCAATGCATTTTTCATAACGTTTTTCCAG
E675Q Forward	GTGGTGCTGCTGCTGCAGTATATTCCGGAAATTACCCTGCCGGTGATTGC
E675Q Reverse	GCAATCACCGGCAGGGTAATTTCCGGAATATACTGCAGCAGCAGCACCAC
E675Q E679Q Forward	GTGGTGCTGCTGCTGCAGTATATTCCGCAGATTACCCTGCCGGTGATTGC
E675Q E679Q Reverse	GCAATCACCGGCAGGGTAATCTGCGGAATATACTGCAGCAGCAGCACCAC
E675K E679K Forward	GTGGTGCTGCTGCTGAAATATATTCCGAAAATTACCCTGCCGGTGATTGC
E675K E679K Reverse	GCAATCACCGGCAGGGTAATTTTCGGAATATATTTCAGCAGCAGCACCAC
